# Lensless magneto-optical imaging

**DOI:** 10.1038/s41598-025-10005-1

**Published:** 2025-08-02

**Authors:** V. Neu, G. Pedrini, I. Soldatov, S. Reichelt, R. Schäfer

**Affiliations:** 1 Leibniz Institute for Solid State and Materials Research Dresden, 01099 Dresden, Germany; 2 Institute of Applied Optics (ITO), University of Stuttgart, 70569 Stuttgart, Germany; 3 Institute of Materials Science, Dresden University of Technology, 01062 Dresden, Germany

**Keywords:** Materials science, Optics and photonics, Physics

## Abstract

Magneto-optical methods, which utilize the interaction of polarized light with the magnetization of the sample in reflection through the magneto-optical Kerr effect or in transmission through the accordant Faraday effect, present prominent and widespread optical microscopy techniques for studying magnetic microstructures. In non-magnetic light microscopy, several alternatives to lens-based imaging have been developed, which offer various advantages, including an improved ratio of field-of-view to magnification. Selected lensless methods also provide access to both intensity and phase information of the probing light field, which presents an additional information channel obtainable from the studied sample. In a proof-of-principle study we verify that the reconstructed magneto-optical intensity obtained from a lensless multiplane recording scheme is in full qualitative agreement with conventional lens-based Faraday microscopy. The additional phase information, not accessible with conventional methods, offers direct access to domain information through the imaginary part of the Faraday or Kerr component in the studied material and allows domain imaging even in a crossed analyzer position or without the use of an analyzer. These findings will open the path to exploit the various established advantages of lensless microscopy for the magneto-optical investigation of magnetic materials.

## Introduction

Resolving magnetic microstructures by magnetic imaging techniques is the key to understanding of both fundamental magnetic phenomena and global magnetic behavior. It allows bridging the internal atomistic order of ferri-or ferromagnetic states and the final functionality of a magnetic material or device on a mesoscopic scale^1,2^. Advanced imaging techniques exist for resolving magnetic microstructures at various length scales, from highly resolving XMCD (X-ray Magnetic Circular Dichroism), Lorentz-type TEM (Transmission Electron Microscopy) or sp-STM (spin-polarized Scanning Tunneling Microscopy) on the sub-10 nm scale to secondary or backscattering electron contrast on the millimeter scale^2^. Magneto-optical methods, which utilize the interaction of polarized light with the magnetization state of the sample in reflection through the magneto-optical Kerr effect (MOKE) or in transmission through the accordant Faraday effect present the most prominent and widespread techniques on an intermediate length scale. Kerr and Faraday microscopy can operate in a wide range of conditions (from cryogenic to high temperature, at variable field strengths, in static, dynamic or stroboscopic fashion) and offer resolution down to a few hundred nanometers, depending on the numerical aperture (NA) of the objective lens and the wavelength of the light^3^. For a recent review on magneto-optical microscopy see^4^. As the size of the lens is typically limited, the field of view (FoV) reduces with high magnification to typically some tens of micrometers.

In non-magnetic light microscopy, several alternatives to lens-based imaging have been developed in the past. Some of them provide access to both intensity and phase information of the reflected or transmitted light wave that can be used for phase imaging and focusing. There are also lensless techniques that offer advantages in terms of the ratio of FoV and magnification^5^. With special phase-retrieval techniques, wave-based imaging is possible without lenses, which first of all removes the need of an additional optical component and furthermore extends the use of light-based imaging techniques to wavelengths, for which classical lenses are no longer available (deep UV, EUV, X-ray)^6^. The most direct phase retrieval method for a light-wave interacting with a sample is holography, which utilizes the interference of the sensing wave-front with a reference beam^7^. Holography allows encoding amplitude and phase information in an interference pattern. With the advance of digital detectors and computing technology, it was possible to record the holograms digitally and process them afterwards^8,9^, which enables direct access to the quantitative phase information. This method, however, often implies severe geometrical restrictions. Other methods do work without a reference beam, and are based on an oversampling or redundancy of data and a subsequent numerical algorithm which determines a self-consistent set of amplitude and phase^10,11^ of both the measured sample and the applied probe, i.e. the plane wave used for illumination. Examples are ptychographic methods^12,13^, which record multiple diffraction images in a fixed plane behind (in case of transmission) or in front (in case of reflection) of the sample with mutual overlap for the required redundancy, or (single pattern) coherent diffraction imaging, which relies on a reference region, typically the known support of the sample^14^. Another, more simple scheme is based on a multiplane recording at various distances along the optical axis^15,16^. All these techniques have meanwhile proven to achieve a resolution very close to or even below the wavelength of the optical light, but at a field of view (FoV) much improved compared to lens-based microscopy^17,18^.

Lensless techniques at visible light frequencies have been applied to a variety of questions, ranging from studying largely transparent, biological samples^19^ to analyzing optically anisotropic materials^20^. However, the measurement of magnetic contrast by lensless optical microscopy has not yet been reported, despite a number of interesting envisioned advantages. Apart from the expected improvement in FoV, the neglect of the objective lens will remove an ever-present artefact encountered in Kerr microscopy, namely the large non-linear intensity background resulting from the Faraday effect in the lens, when a magnetic field is applied to the sample (and typically also to the objective lens). Furthermore, intensity and phase reconstruction provide complete information about the scattered light field that is not accessible with conventional lens-based methods, and can be used for phase imaging and focusing. Last but not least, without the need of an objective lens, magneto-optical imaging might be easier in the deep UV range, immediately allowing for an improved resolution.

At much shorter wavelengths, i.e. for X-ray-based magnetic imaging, lensless techniques (ptychography, coherent diffraction imaging and holography) are meanwhile well established^21,22^. There, the magnetic contrast mechanism relies, however, on the helicity-dependent absorption of circularly polarized X-ray photons at core level electron transitions. The objective of this work is to verify the feasibility of lensless magnetic imaging in the optical regime, where the interaction of linearly polarized light with the magnetization $\overrightarrow{m}$ of the sample results in a rotation of the polarization plane and a possible occurrence of ellipticity, known as magneto-optical Kerr effect in reflection and Faraday effect in transmission. As a proof-of-principle study we do not aim at imaging results superior to conventional lens-based Kerr or Faraday microscopy, but rather explore and demonstrate the different contrast mechanisms that can be applied in a lensless approach. By comparing reconstructed intensity images with the expectation of conventional imaging, we can validate the soundness of the lensless method, and by analyzing phase images we are able to validate the added value, which the lensless method offers through the phase reconstruction. We restrict ourselves to optically transparent magnetic garnet films, i.e. just applying the Faraday effect. In such films the magnetic domains are well known and understood^2^ so that the interpretation of our imaging data is not complicated by unknown aspects of the domain patterns.

## Results and discussion

**Fig. 1:**
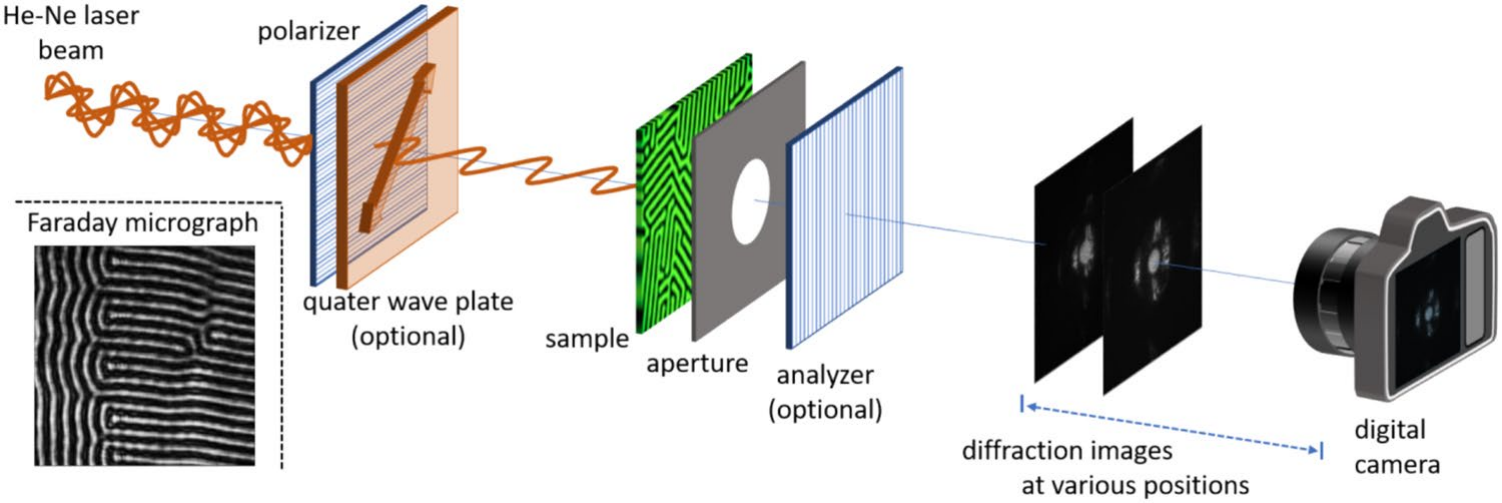
Schematics of the transmission multiplane setup for lensless magneto-optical imaging. The linearly polarized light of a He–Ne laser is transmitted through the sample, an aperture of 500 μm diameter and an optional analyzer, and diffraction images at selected positions behind the sample are recorded. Alternatively, a quarter wave plate behind the polarizer converts the linearly polarized light into circularly polarized light. The inset (lower left) displays a polar Faraday micro-graph (150 μm × 150 μm) of the perpendicular domains of the garnet sample recorded in a conventional lens-based approach.

The sample studied in these proof-of-principle experiments is a doped Yttrium-Iron Garnet (YIG) film with perpendicular magnetic anisotropy grown on a transparent Gadolinium–Gallium Garnet (GGG) substrate and provided by M. Lindner from Innovent e.V. In zero field, the magnetization forms a demagnetized band domain state with balanced domain widths of about 4.5 µm, as seen in a conventional Faraday microscopy image of the sample (Fig. 1, inset). The dark and bright bands correspond to regions of homogeneous magnetization (domains) oriented perpendicular to the surface in either up or down direction. All lensless experiments are performed on that sample in a transmission multiplane imaging setup sketched in Fig. 1 and documented in the Supplementary Fig. S1. Further details are given in the methods section.

### Magneto-optical imaging with linearly polarized light and almost crossed analyzer

**Fig. 2:**
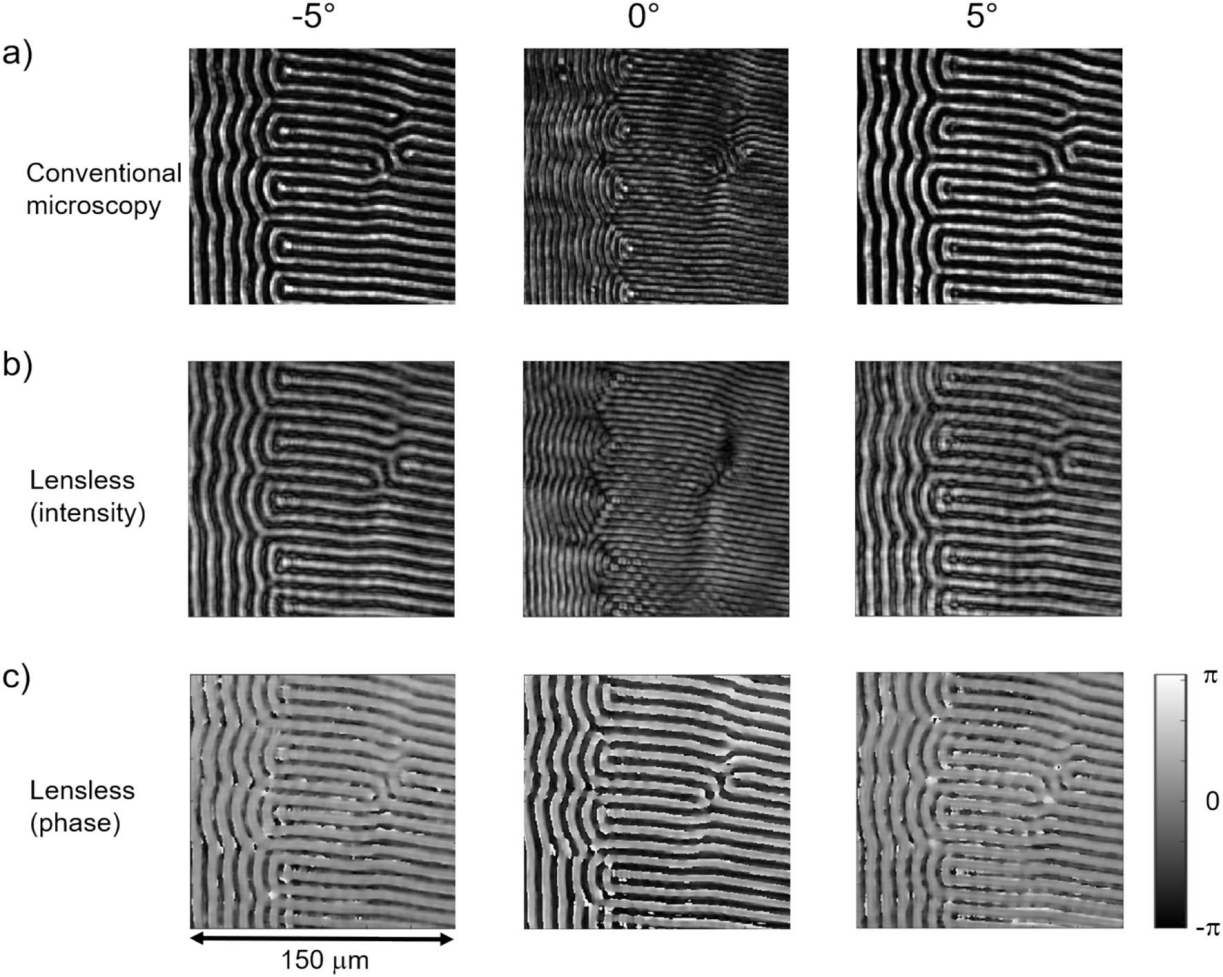
Magneto-optical investigation with linearly polarized light and analyzer. (**a**) Magneto-optical image of the garnet sample recorded by conventional lens-based microscopy. (**b**) Intensity and (**c**) phase reconstructed from measured diffraction planes in the lensless setup. The measurements used an analyzer on top of the sample at the crossed position (90°) and a linear polarizer set to − 5°, 0° and + 5°.

Figure 2a displays conventional lens-based magneto-optical images of the garnet sample (zoom from the 500 µm diameter FoV defined by the pinhole), obtained with a similar experimental setup as for the lensless approach, but with the digital camera replaced by a microscope (objective lens 10×, NA = 0.25), that records the image by a CMOS sensor (see Supplementary Information, Fig. S2). The analyzer was in the crossed position (90°) with respect to a polarizer angle of 0° and the images were taken at polarizer angles of − 5°, 0° and + 5°. The interaction of the polarized light $\overrightarrow{E}$ with the magnetization $\overrightarrow{m}$ of the sample can be described as an additional complex (Faraday) field vector $\overrightarrow{F}$ that adds to the normally transmitted field $\overrightarrow{E}$-field, which is polarized along the same direction as the incoming light. The transmitted field is then given by $\overrightarrow{{E'}}=\overrightarrow{E}+\overrightarrow{F}$, with $\overrightarrow{F}$ being expressed through the material dependent Voigt constant ${Q}_{V}$ as $\overrightarrow{F}=i{Q}_{V}\ (\overrightarrow{m}\times \overrightarrow{E})$  (ref. ^2^). In the present simple geometry (out-of-plane magnetization and electric field vector $\overrightarrow{E}$ parallel to the plane of the sample), the Faraday component $\overrightarrow{F}$ stays within the plane of the sample, is perpendicular to the incoming $\overrightarrow{E}$ vector and changes orientation with the polarity of the magnetization.

The real part of $\overrightarrow{F}$, which is in-phase with the incoming vector $\overrightarrow{E}$, leads to a polarization rotation of the transmitted light $\overrightarrow{{E'}}$, the imaginary part with its 90° phase shift will result in an elliptical distortion. Due to the dependence on $\overrightarrow{m}$, the polarization plane of the light will rotate in opposite directions when passing through oppositely magnetized polar domains. For a given non-zero polarizer angle (e.g. − 5°) the analyzer blocks the two rotation directions differently (note that the Faraday rotation angle is much smaller than the opening angle of the analyzer typically used in magneto-optical microscopy), leading to a visualization of the domain pattern as alternating large (bright) and low (dark) intensity. With the polarizer being set to the opposite side of the crossed position (+ 5°), now the oppositely rotated light is predominately blocked, essentially inverting the contrast. In the fully crossed position (0°), an optical diffraction effect occurs, which stems from the interference of light at the domain boundaries, and which is also known in conventional Faraday microscopy^23^. While the intensity parallel to the polarization axis is fully attenuated for crossed polarizer and analyzer, the small perpendicular component resulting from the polarization rotation can pass the analyzer, though with identical amplitude for the two domain states and hence no expected intensity contrast. These amplitudes, however, do have opposite signs, i.e. the light field from neighboring domains has a 180° phase shift, which leads to a destructive interference within a region around the domain boundary that is determined by the resolution of the optical system. Consequently, the domain boundaries are imaged as regions of reduced intensity or dark lines as seen in the central image of Fig. 2a.

Figure 2b,c summarize the reconstructed intensity and phase of the *lensless* magneto-optical imaging approach. As in the conventional case (Fig. 2a), one clearly identifies the two polar domain orientations in the intensity channel if the polarizer is set away from the crossed orientation, and upon inverting the polarizer position the contrast inverts. Also in the fully crossed position (0°), the intensity image corresponds to that of the conventional, lens-based image. It differs only slightly in the width of the destructive interference regions (dark lines), which is increased as compared to conventional imaging. A direct line profile comparison of lens-based and lensless approach across the intensity images taken at − 5° polarizer setting (Supplementary Information, Fig. S3) confirms the overall qualitative agreement, but also reveals some distortions aside the intensity minima of the lensless image. We do attribute this slightly inferior image quality in the lensless approach to reflections within or between different elements of the setup (sensor, protective glass, analyzer, sample) creating unwanted interference patterns when they overlap with the light diffracted by the sample. In addition to providing the intensity, the lensless approach now also allows reconstructing the phase shift of the polarized light interacting with the domains of opposite out-of-plane polarity. We observe a clear phase contrast for oppositely magnetized domains in agreement with an imaginary part of the Faraday component, which furthermore inverts, when changing the polarizer angle from − 5° to + 5°. Of especial significance is the phase image at the fully crossed polarizer setting (central image in Fig. 2c), which in contrast to the intensity images, now directly presents the domain pattern like a regular polar Faraday image. This is in full accordance with the 180° phase shift between the two oppositely oriented Faraday components of identical amplitudes which pass the fully crossed analyzer.

Overall, the lensless approach not only preserves the same contrast mechanism in the intensity channel as standard Faraday (or Kerr) microscopy, but also produces a clear domain contrast in the phase channel, particularly for polarizer settings near or at the crossed orientation. This will e.g. offer advantages for imaging magnetic materials with predominantly phase-shifted Faraday (or Kerr) components. Then the intensity contrast, which relies on different blocking of the two rotated polarization directions will be subdued for a reduced in-phase Faraday component (and thus Faraday rotation), whereas the ± 90° phase shift is still accessible through the phase reconstruction of the light field.

### Magneto-optical imaging with linearly polarized light and without analyzer

**Fig. 3:**
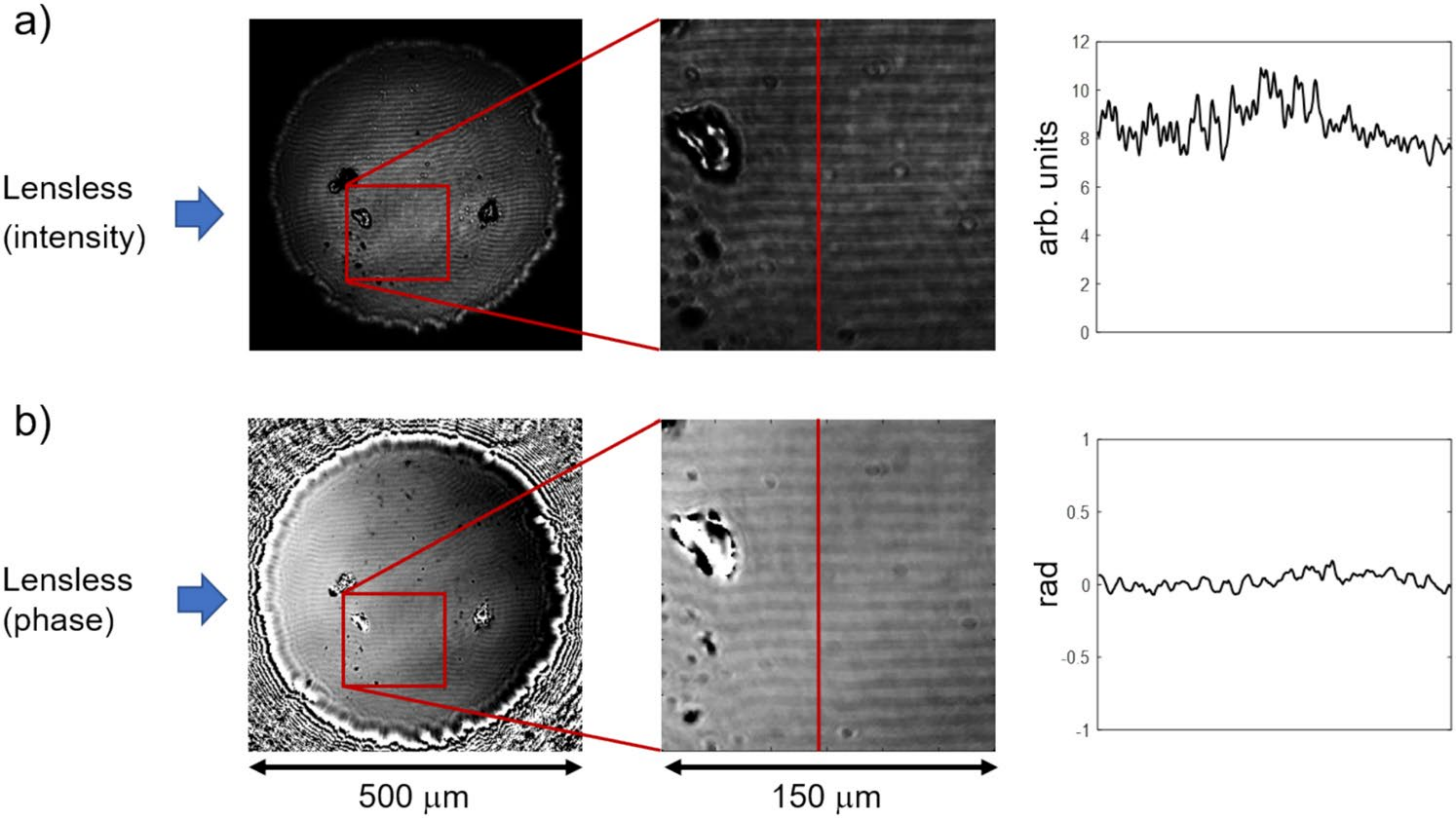
Magneto-optical investigation with linearly polarized light without the use of an analyzer. (**a**) Intensity and (**b**) phase reconstructed from measured diffraction planes in the lensless setup are shown as images from the total field of view (left), as a selected region of 150 μm × 150 μm (middle), and as a contrast profile across the clearly visible band domains (right). Note, that the contrast in the images is enhanced for better visibility. The absolute values e.g. of the phase contrast (≈ 0.1) are much smaller than in Fig. 2, where a maximum phase contrast of π is observed.

This latter observation suggests, that domain contrast is observable through the phase channel even in absence of an analyzer. The following experiments are thus performed with the polarizer nominally set to the 0° position, but the analyzer removed. Figure 3 displays the reconstructed intensity (a) and phase channel (b). Indeed, the band domains are well visible in the phase channel, but with a much smaller contrast than in case of the crossed analyzer. Differently from the previous experiment the regularly transmitted light field $\overrightarrow{E}$ is not suppressed by the analyzer, and the contrast in the phase shift might relate to the relative proportion of the Faraday component to the regular component. Interestingly, also the reconstructed intensity image shows a contrast variation according to the sample’s domain structure, although the amplitude of the transmitted light should not depend on the polarity of the perpendicular Faraday component. There may be several reasons for this, which we need to investigate further: One possibility is an unintended mixing of phase and intensity channel in our reconstruction algorithm, which needs to be investigated in future work. Other possibilities relate to similar unexpected observations in conventional lens-based Kerr and Faraday experiments without analyzer. *Lambeck*^23^ already reported domain boundary contrast in an iron film, comparable to that observed for a fully crossed analyzer, but now running the polarization microscope in the dark field mode and without an analyzer. Similarly^24^, a very weak domain boundary contrast was observed in analyzer-free polar Kerr microscopy on a garnet film with perpendicular anisotropy (note that the Kerr microscope was effectively used as a Faraday microscope by placing a metallic mirror on the backside of the transparent specimen). In that case, the contrast was even dependent on the polarizer angle and changed to a weak domain contrast for a polarizer angle of 45° with respect to the tilt axis of a mirror in the beam path of the Kerr setup. The authors offered an explanation for the latter finding by anticipating the generation of elliptically polarized light introduced by the mirror, which then results in a domain contrast due to the effect of circular magnetic dichroism. In our present setup, with a linear polarizer behind a 45° mirror (see Fig. S1 in the Supplementary Information), this explanation can, however, not hold. While the appearance of intensity contrast in the present imaging configuration (linear polarized light transmitting with normal incidence through a magnetic sample with perpendicular domains) is thus still not settled, disentangling phase and intensity contrast in the lensless approach may offer an explanation in the future.

### Magneto-optical imaging with circularly polarized light and without analyzer

The magneto-optical Faraday (and Kerr) rotation and ellipticity may be interpreted as circular magnetic birefringence and dichroism, respectively. According to this Fresnel concept, a linearly polarized light wave must be seen as being composed of two circularly polarized partial waves of opposite handedness and equal amplitude. While propagating in the material, both partial waves feel different indices of refraction due to the magnetization so that they propagate with different velocities thus being phase-shifted (called circular birefringence). The emerging linear wave, being composed of the two phase-shifted circular waves, is then rotated (Faraday or Kerr rotation). If there is also different, magnetization-dependent damping of the two circular waves, magnetic circular dichroism will lead to superimposed Faraday (or Kerr-) ellipticity^25^).

**Fig. 4:**
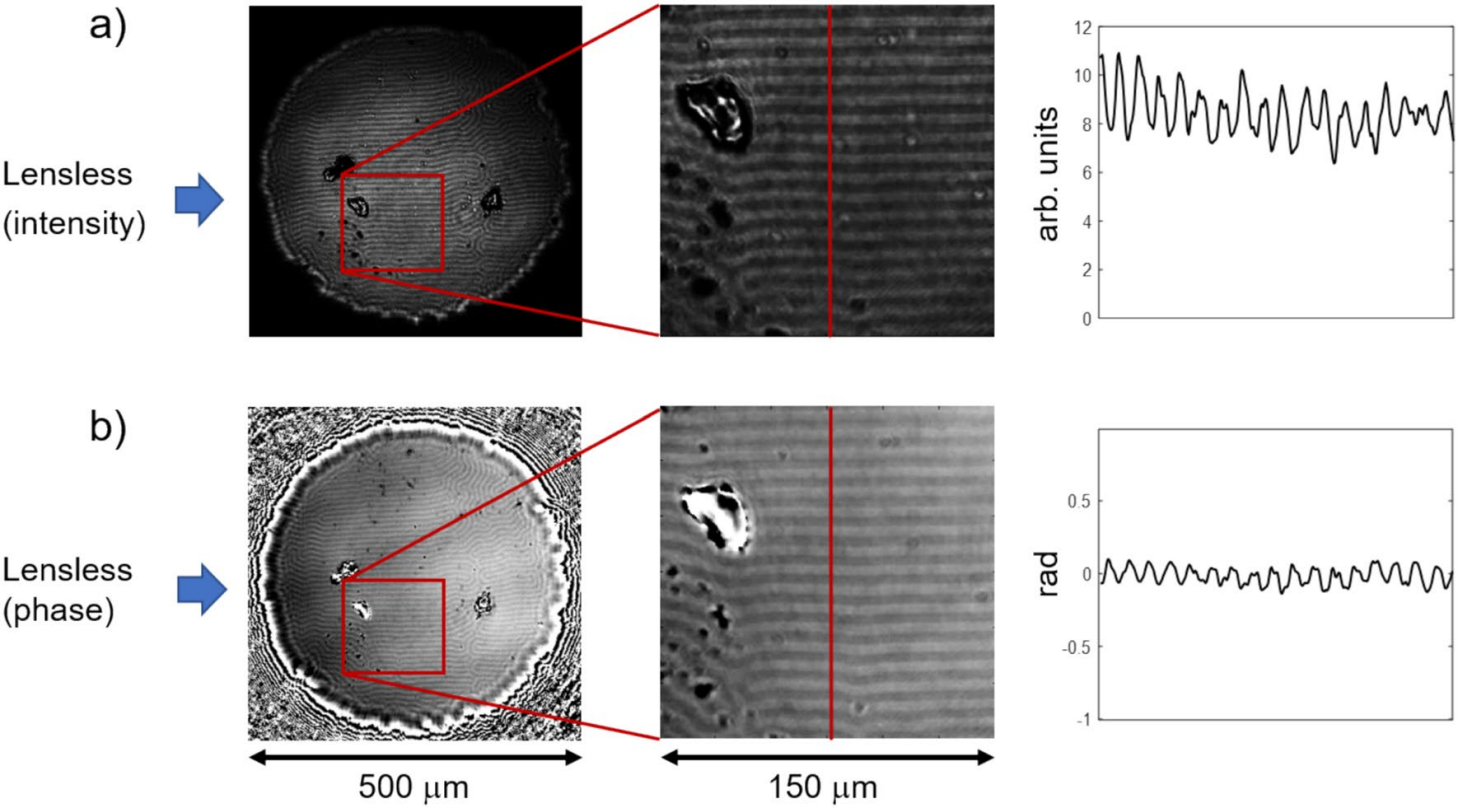
Magneto-optical investigation with circularly polarized light without the use of an analyzer. (**a**) Intensity and (**b**) phase reconstructed from measured diffraction planes in the lensless setup are shown as images from the total field of view (left), as a selected region of 150 μm × 150 μm (middle), and as a contrast profile across the clearly visible band domains (right). Note, that the contrast in the images is enhanced for better visibility. The absolute values e.g. of the phase contrast (≈ 0.2) are much smaller than in Fig. 2, where a maximum phase contrast of π is observed.

A simpler situation occurs, when the domain pattern is intentionally illuminated with circular light of just one handedness in normal incidence. This is realized by adding a quarter wave plate after the polarizer in the beam path, before the light transverses the magnetic sample. Then a Faraday (or Kerr-) rotation, based on circular birefringence, is no longer possible, but circular dichroism is, leading to a different damping of the circular wave for different magnetization directions. Hence, even without an analyzer, the circular magnetic dichroism will lead to a polarization dependent amplitude change of the light resulting in an intensity contrast as seen in Fig. 4a. This effect, which is well explored in X-ray based absorption experiments of core-level excitations^25^, has its analogy at optical frequencies and has lately been rediscovered to reveal intensity contrast in magnetic thin films with perpendicular domains^24,26,27^. As demonstrated in our experiments it can likewise be harvested in a lensless optical setup and offers a well visible contrast. Furthermore, the reconstructed phase (Fig. 4b) reveals a similarly well-defined domain contrast, now based on the circular magnetic birefringence effect. With the light velocity being modified by the sample magnetization, the circularly polarized light acquires a phase shift in opposite directions when propagating through the two oppositely magnetized domains.

## Conclusions

In a set of three multiplane recording experiments, we demonstrated that the reconstructed intensity obtained from this lensless magneto-optical imaging approach is in full qualitative agreement with the intensity contrast observed and expected from conventional lens-based Faraday microscopy. This holds for the usual application of linearly polarized light with an almost crossed analyzer, but also for the less common combination of linearly polarized light with fully crossed analyzer and for measurements with circularly polarized light without analyzer. In the case of linearly polarized light without analyzer, the unexpected appearance of intensity contrast still needs to be investigated further. The additional phase information, not accessible with conventional lens-based methods, offers direct access to domain information in a crossed analyzer setup, irrespective of the magnitude of the polarization rotation. Furthermore, as the phase shift of the light field is directly related to the imaginary part of the magneto-optical component in the studied material, the phase retrieval offers advantages for imaging magnetic materials with predominantly phase-shifted Faraday (or Kerr) components. In total, in this work the ptychographic magnetic imaging established for x-ray absorption has been shown to also work in the visible spectrum via the magneto-optical effect. This initial verification of lensless magneto-optical imaging will enable the various established advantages of lensless microscopy to be utilized for the examination of magnetic materials in the future.

## Methods

### Experimental setup

For the reconstruction of intensity and phase contrast of the imaged sample, the lensless multiplane recording method was used^15,16,18^ The experimental setup described in^18^ was modified to allow the investigation of magnetic microstructures (see Fig. S1). A non-polarized He–Ne laser beam with a wavelength of 633 nm is reflected by a mirror towards the sample. A linear polarizer allows for the choice of linear polarization orientation of the light incident on the sample. For applying circularly polarized light, a quarter-wave plate was positioned behind the polarizer. Furthermore, a circular aperture (diameter 500 µm) was placed above the sample, to limit the Field of View. This is necessary in order to restrict the high spatial frequencies on the sensor, which could lead to aliasing, according to the Nyquist-Shannon sampling theorem^28^. An additional advantage of using this small pinhole is that it reduces the overlap between the diffraction pattern and unwanted reflections generated by various elements of the setup (sensor, protective glass, analyzer, sample). This allows some of the reflections to be filtered out without excessively disturbing the diffraction pattern. For some experiments, an analyzer is inserted after the aperture. The light diffracted by the sample is recorded by a CMOS sensor mounted on a Physik Instrumente M-126 translation stage having a displacement accuracy of 2.5 µm. We take *N*_*z*_ = 40 measurements spaced by Δ*z* = 0.1 mm, for a total sensor travel distance of *z*_*T*_ = 4.0 mm. The closest and farthest recording planes are at 4.5 mm and 4.5 + 4.0 = 8.5 mm from the target. These are optical distances calculated from the measurements and they differ slightly from the physical distances due to the refractive index of the camera protective glass. A Matlab program is used to drive the translation stage and to collect data from the camera. The sensor is a monochromatic board level CMOS camera (Basler, daA3840-45um), i.e. it has no housing such that one can move it very close to the sample. It has a resolution of 3840 × 2160 pixels and a pixel size of Δ*x* × Δ*y* = 2 µm × 2 µm.

### Data acquisition

For all measurements we first crop the camera output to *N*_*x*_ × *N*_*y*_ = 2048 × 2048 pixels. Collecting light with high dynamic range is important in lensless imaging, for this reason the diffraction patterns were recorded with two integration times. The lower integration time is chosen to avoid saturation of the sensor. The second integration time is 16 times larger. At each plane we first perform 10 measurements at the large integration time, average the results and keep the unsaturated pixels. The values for the saturated pixels are taken from 10 averaged measurements at low integration time, multiplied by a factor of 16. By using a 12-bit sensor we thus obtain an effective dynamic range of 16 bits (12 bits from the measurements taken at higher integration time + 4 bits from the measurements taken at lower integration time and multiplied by 16 = 2^4^).

### Data reconstruction

The reconstruction algorithm is based on the experimental amplitudes at the 40 recorded positions (planes) along the optical axis, which are calculated as the square root of the measured intensities. The unknown phase is successively refined by adding an estimated phase to the amplitude at a given plane and propagating the such obtained complex wave front via the angular spectrum method^29^ plane by plane from this plane to all the others. At all those planes the calculated (propagated) amplitude is replaced by the measured one, and the calculated phase becomes the new estimate for this plane. One iteration corresponds to propagating through all the 40 planes in a random order. For a complete reconstruction, 200 iterations are performed, always with a new random permutation of the order in which the planes are visited.

As the algorithm processes discrete data, the pixel size of the sensor (2 × 2 µm^2^) presents a resolution limiting factor, which can however be mitigated by pixel splitting. After a full iteration, each pixel is split into 4 quarters (now of size 1 × 1 µm^2^) and the data are iterated again. As the initial guess for the new round of iterations, each of these four sub-pixels receives the same amplitude and phase as the original pixel that was split. This step is repeated for another pixel splitting, now with subpixel size 0.5 × 0.5 µm^2^. This improves the potential resolution of the algorithm to beyond the expected resolution of 1.25 µm based on the numerical aperture (see Supplementary Information) The reconstruction algorithm is described in more detail in reference^18^.

## Supplementary Information

Supplementary Information.

## Data Availability

The datasets generated and analyzed during the current study are available from the authors on reasonable request. Point of contact: Dr. G. Pedrini, giancarlo.pedrini@ito.uni-stuttgart.de.
