# Supplementary material for: Lensless magneto-optical imaging
Source: Sci Rep. 2025 Aug 2;15:28277. doi: 10.1038/s41598-025-10005-1 (PMC12317973; doi:10.1038/s41598-025-10005-1)
Supplement: Supplementary file 1 — Supplementary Information. [file 41598_2025_10005_MOESM1_ESM.docx]

**Lensless magneto-optical imaging**

V. Neu^1^, G. Pedrini^2^, I. Soldatov^1^, S. Reichelt^2^, R. Schäfer^1,3^

^1)^ Leibniz Institute for Solid State and Materials Research Dresden, D-01099 Dresden, Germany

^2)^ University of Stuttgart, Institute of Applied Optics (ITO), D-70569 Stuttgart, Germany

^3)^ Dresden University of Technology, Institute of Materials Science, D-01062 Dresden, Germany

1) Lensless magneto-optical setup


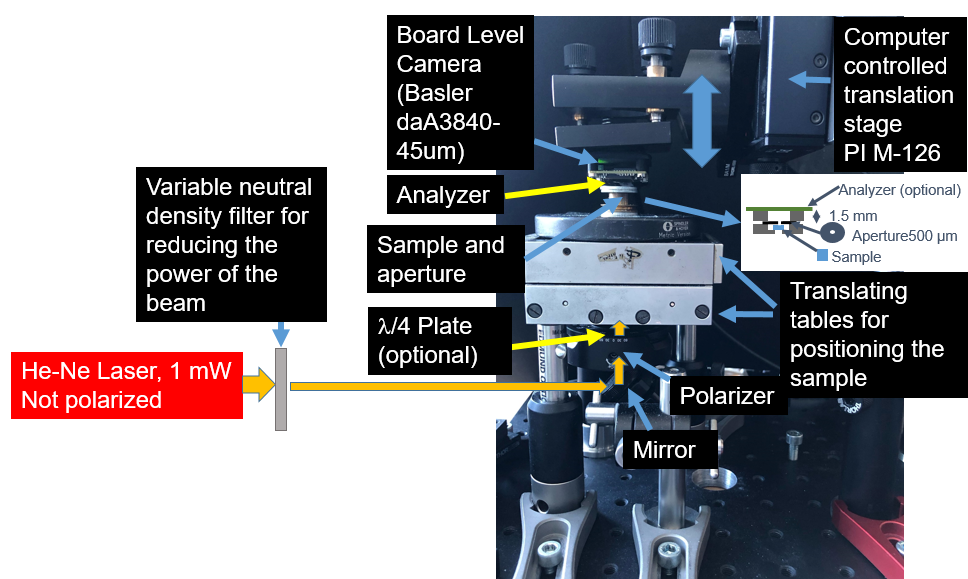


Figure S1: Lensless magneto-optical setup

Figure S1 shows the setup used for the investigations. A He-Ne laser having wavelength 633 nm (NEC GLG5002, not shown in the figure) provides the illumination in a horizontal beam. The intensity can be adjusted via a variable neutral density filter. The light hits a 45° mirror, which redirects the beam into the vertical direction. By means of a polarizer (Thorlabs RSP1D/M) the light illuminating the sample will be linearly polarized and the polarization orientation can be selected, with 0° corresponding to a polarization orientation normal to the plane of the figure. In some experiments a quarter wave retarder (Linos retardation film, wavelength range 450 - 650 nm) was inserted right after the polarizer for generating light with circular polarization. The light continues towards a translational sample stage, on which the sample, the aperture and the analyzer (optional) are mounted by means of a mechanical support. This support is built from simple mechanical components (washers, bolts), joining the sample, the 500 µm diameter aperture (heavy stock black paper, cut with a laser cutter) and the analyzer in a geometry sketched in the inset of Fig. S1. The analyzer (cut from a 2" x 2" dichroic film sheet, Thorlabs) is oriented such that it is fully blocks the unperturbed linearly polarized light, when polarizer position is set to 0° (crossed geometry)

A monochromatic board level CMOS camera (Basler AG, model daA3840-45µm) is mounted on a vertical translational stage (PI M-126), which is moved along an axis parallel to the illuminating laser beam. As the camera has no housing, it can be brought as close as 2 mm to the sample support, which corresponds to a minimum of 4 mm with respect to the upper sample surface.

2) Lens-based magneto-optical setup


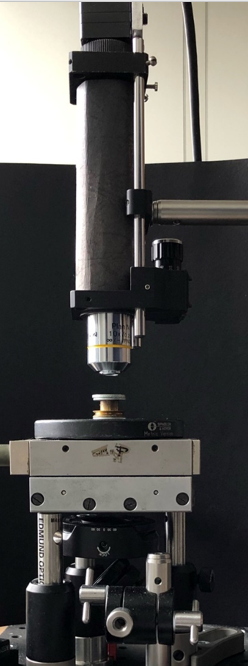


Figure S2 shows the conventional setup used, in which the sample is imaged with a microscope objective (10x, NA=0.25) on a CMOS sensor (XI-MEA, xiQ, model MQ013MG, 1280 x 1024 pixels, pixel size 5.3 µm). The sample is illuminated with light of different polarizations in a similar way as for the lensless setup shown in Figure S1. In some experiments, an analyzer (not shown in Figure S2) was inserted between the sample and the microscope objective.

Figure S2: Lens-based magneto-optical setup

3) Line-profile comparison


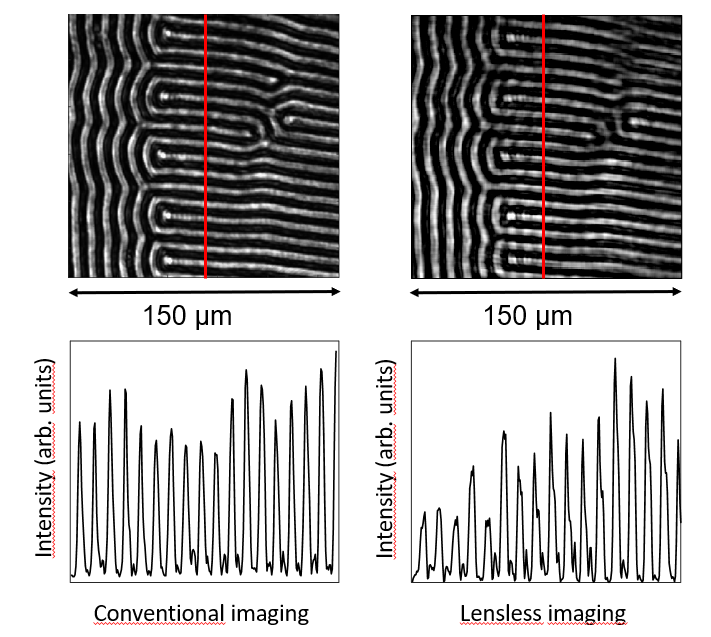


Fig. S3: Line profiles of intensity images taken at a polarizer setting of -5° and an analyzer at 90°

Figure S3 displays line profiles of intensity image taken at a polarizer setting of -5° and an analyzer at 90°, across the same domain region, showing overall qualitative agreement in the appearance of positive and negative domains. The intensity is given in arbitrary units and is thus purely qualitative. One has to recognize, however, that except for special quantitative approaches, magneto-optical measurements recording the intensity of transmitted or reflected light are anyway purely qualitative. The measured intensity does depend so delicately on the optical conditions (camera settings, illumination, beam path, …), that a quantitative intensity comparison is impossible for different optical setups. Furthermore, the profiles reveal distortions aside the intensity minima in the lensless imaging. We do attribute this to reflections within or between different elements of the setup (sensor, protective glass, analyzer, sample) creating unwanted interference patterns when they overlap with the light diffracted by the sample. Some of the interference patterns generated by reflections are stationary, while others change during the acquisition process due to the relative movement of the reflecting elements. The impact of unwanted interference on the quality of the reconstructed image is difficult to quantify. There are various ways to prevent reflections. These include removing the sensor's protective glass, applying an anti-reflective coating, or using a short-coherence light source. The implementation of these reflection reduction techniques will be the subject of future research.

4) Estimated resolution

Pixel-splitting was used to increase the resolution beyond the size of the sensor pixels, such that the resolution is expected to be diffraction-limited and given by the wavelength of the light and the numerical aperture (NA) via $R=0.82\cdot\lambda/{NA}$ [1]. In our experiment $NA=\sin(\tan^{-1} (L/{2z}))$, where *z* and *L* are the sample-sensor distance and the size of the sensor, respectively. Considering a distance *z* = 4.5 mm (the resolution is determined by the (first) recording plane closest to the sample [18] and a sensor size *L* = 4.096 mm (2048x2 µm), the numerical aperture is calculated as *NA* = 0.42 and the resolution is *R* = 1.25 µm.
